# The necessity of connection structures in neural models of variable binding

**DOI:** 10.1007/s11571-015-9331-7

**Published:** 2015-02-10

**Authors:** Frank van der Velde, Marc de Kamps

**Affiliations:** 1 Technical Cognition, CPE-CTIT, University of Twente, P.O. Box 217, Enschede, 7500 AE The Netherlands; 2 IO, Leiden University, Leiden, The Netherlands; 3 Biosystems Group, School of Computing, University of Leeds, LS29JT Leeds, UK

**Keywords:** Behavior, Frame of reference, Small-word networks, Novel variable binding

## Abstract

In his review of neural binding problems, Feldman (Cogn Neurodyn 7:1–11, 2013) addressed two types of models as solutions of (novel) variable binding. The one type uses labels such as phase synchrony of activation. The other (‘connectivity based’) type uses dedicated connections structures to achieve novel variable binding. Feldman argued that label (synchrony) based models are the only possible candidates to handle novel variable binding, whereas connectivity based models lack the flexibility required for that. We argue and illustrate that Feldman’s analysis is incorrect. Contrary to his conclusion, connectivity based models are the only viable candidates for models of novel variable binding because they are the only type of models that can produce behavior. We will show that the label (synchrony) based models analyzed by Feldman are in fact examples of connectivity based models. Feldman’s analysis that novel variable binding can be achieved without existing connection structures seems to result from analyzing the binding problem in a wrong frame of reference, in particular in an outside instead of the required inside frame of reference. Connectivity based models can be models of novel variable binding when they possess a connection structure that resembles a small-world network, as found in the brain. We will illustrate binding with this type of model with episode binding and the binding of words, including novel words, in sentence structures.

## Introduction

Feldman (2013) reviewed a number of solutions of neural binding problems as reported in the literature. Neural binding problems arise when information is processed in different neural circuits, distributed over the brain. One example is the visual processing of shape (e.g., the identification of a face), which begins with the detection of oriented lines in the primary visual cortex. Because a face is more than a collection of oriented lines, the question arises how the processing of these lines is recombined into the recognition of the face. Other examples in vision are the binding of visual features of an object (e.g., its shape, color, motion, location), which are also (partly) processed in different brain areas. Outside vision, binding problems arise, for example, in language processing. Sentences consist of words and words consist of phonemes and morphemes. Again the question is how these different parts of information are combined.

The examples given already show that binding problems can arise in different domains. In his review Feldman aimed to reduce the confusion in discussing the binding problem by distinguishing between binding problems arising in coordination, the subjective unity of perception, visual feature binding and variable binding (e.g., binding words in a sentence structure). It is indeed a sensible approach to distinguish between different forms of the binding problem.

However, Feldman (2013) did not reduce the confusion in this treatment of (novel) variable binding. In this section, Feldman primarily compared two types of models. One of those he referred to as ‘static’ models. In these models, variable binding depends on a given (available) connection structure. The other type of models could be referred to as ‘label’ models. In these models, binding seems to be achieved by marking the activation of the variables to be bound (e.g. *John* as the agent of *loves* in *John loves Mary*) with some (changeable) label, such as synchrony of activation.

The best known model of binding by synchrony, according to Feldman, is Shruti (Shastri and Ajjanagadde 1993). Hence, Feldman used binding by synchrony in Shruti to illustrate variable binding, including novel variable binding, such as the binding of (novel) items in novel sentences or propositions. In Feldman’s view ‘static’ models are inadequate for novel variable binding, because it would seem that novel variable bindings would require novel connection structures, which are not available at that moment. So, label models as given by synchrony of activation, such as Shruti, would be the only viable candidates in Feldman’s view.

We will show that Feldman’s analysis of novel variable binding in both types of models is incorrect. We will show that models based on available connection structures (i.e., ‘static’ models in Feldman’s terminology) are the only viable candidates for neural models of variable binding, including novel binding. Label (synchrony) based models are either specific examples of models based on available connection structures, or they are, in a fundamental way, inadequate as models of neural binding.

Examples of models based on available connection structures are models of episode binding in the hippocampus (Norman and O’Reilly 2003), binding of perception and action (Zylberberg et al. 2010) and models of binding words in sentence structures (van der Velde and de Kamps 2006a, b, 2010). As we will show, the binding process in such models is in fact very dynamical. Hence, we prefer the label ‘connectivity based’ instead of ‘static’ for models depending on existing connection structures.

The reason why connectivity based models *are* the only viable candidates as models of binding derives from the fact that they are the only models that can produce behavior, which is an essential requirement for any neural model of cognition. We will illustrate that synchrony (label) based models indeed reduce to connectivity based models whenever they produce behavior.

The reason why connectivity based models *can be* models for binding derives from the fact that the connection structure of the brain resembles that of a ‘small-world network’ (Watts and Strogatz 1998). Such connection structures provide the flexibility needed for novel variable binding. This is even true for the binding of novel items (e.g., words) in novel structures (e.g., sentences), provided we take the boundary conditions on these forms of binding into account.

We will discuss these issues in turn. We start with the issue of variable binding in novel combinations, because that issue is crucial for the discussion in our paper.

## Variable binding in novel combinations

The issue of novel binding of variables is introduced by Feldman in the following way (2013, p. 7b): “if I tell you that my granddaughter Sonnet is brilliant, you have a new person to consider as a possible filler for variable roles and also a number of new facts for use in inference.” Note the emphasis here on a “new person” and “new facts”. “New person” could be seen as a new name or word, “new facts” could be seen as new propositions. In other words, we have the ability to bind new words in new propositions never seen before, and a binding theory/model would have to account for that in Feldman’s view (and ours). According to Feldman, the fact that we can bind new words in new propositions indicates that we need a label such as synchrony to achieve variable binding. A binding model based on available connection structures would not be able to do this in his view, because the connection structure that integrates the new name in a new proposition would not be available.

Although the possibility of novel variable combinations is just one of the issues related to binding, it is a crucial one because it relates to an important aspect of human cognition. Human cognition is productive in the sense that we can create and understand an unlimited set of variable combinations (e.g., sentences in language).

George Miller, for example, derived a lower bound of this ability by calculating how many sentences could be made of 20 words or less, using a lexicon of the size of human language. He arrived at a set of 10^20^ sentences (see Pinker 1994). By comparison, the life time of the universe since the Bing Bang as estimated by astronomy is in the order of 10^18^ s. So, this ‘Miller set’ of sentences (propositions) is so large that we cannot have a dedicated representation for most of them. Notice in particular that the set of all propositions that is stored in a long term memory, i.e., the set of sentences for which we do have dedicated representations, is only a (very) small subset of the Miller set.

Yet, we can understand any arbitrary sentence from the Miller set, certainly in the sense that we can answer ‘who does what to whom’ questions about such a sentence, even though we could not have encountered most of these sentences before. The ‘Sonnet’ sentence of Feldman, quoted above, is an example (explicitly constructed as such by Feldman). In fact, if ‘Sonnet’ is a new name (word), this sentence is even outside the Miller set, because that is based on an existing lexicon. So, we can even understand novel sentences with new words in it. We could call this the ‘extended Miller set’.

Thus, the ‘Sonnet’ sentence that Feldman used to illustrate the issue of novel binding is a sentence that we have never seen or heard before, but that we can understand. In particular we could answer a question like “who is brilliant?”. The relevance for the issue of variable binding is that most sentences in the (extended) Miller set are of this type (i.e., novel sentences). This raises the question of how binding relations in these novel sentences could be instantiated. As noted, in Feldman’s view this could not be done on the basis of an existing connection structure. Indeed, he used the ‘Sonnet’ sentence to argue that this is not possible. Instead, in his view, it can be done only with synchrony of activation.

But here a problem or confusion arises in his paper. Feldman used the Shruti model (Shastri and Ajjanagadde 1993) as the example to show how synchrony of activation could solve the binding issue in novel sentences. The confusion is that Shruti is in fact a model of inferences in long term memory. To produce these inferences, Shruti critically depends on (entire) proposition representations for its operations (see below for an example). This entails that Shruti cannot be a model for novel binding as found in the ‘Sonnet’ sentence given by Feldman, because there is no proposition representation for this sentence in long term memory (as explicitly ruled out by Feldman himself).

This raises an important question: why does Feldman believe that Shruti can solve the binding issue in novel combinations (as in the ‘Sonnet’ sentence) when in fact it cannot? To answer this question, we first have to illustrate the Shruti model. In particular, we have to show how the model produces behavior (in this case, making inferences in long term memory).

## Connection structures and behavior

A cognitive process is of value to an organism when it can influence its behavior, which implies that it is incorporated in the sensorimotor loop of the organism (Shanahan 2010). That is, a cognitive process somehow transforms sensory information, carried by sensory nerves, into actions of some kind, initiated by the activation of motor neurons. Thus cognitive processes are executed in connection structures that connect sensory circuits with motor circuits (often in a bi-directional way, hence the notion of a loop).

For example, in the ‘Sonnet’ sentence of Feldman (2013) the perception of the name “Sonnet” activates (sensory) neurons. In answering the question “who is brilliant?”, motor neurons are activated that produce the name “Sonnet”. Regardless of how complex such cognitive processes are, underlying connection structures are needed to carry the flow of activation initiated in the senses to the muscles (or vice versa). So, if a cognitive process requires (novel) variable binding, e.g., the binding of items in a combinatorial (compositional) structure (such as words in a sentence), the binding of the items is also somehow incorporated in the neuronal connection path that links perception and action.

We can illustrate this with synchrony based models of binding. Feldman (2013, Fig. 3) illustrated the Shruti inference network (Shastri and Ajjanagadde 1993; Wendelken and Shastri 2004). The inference illustrated concerns the notion that a buyer of an object is also the owner of the object. However, Feldman (2013) presented only a part of the inference network, as do Wendelken and Shastri (2004). The (more) complete circuit, accounting for the production of behavior (the inference in this case) is found in Figure 12 of Shastri and Ajjanagadde (1993). This figure illustrates two closely related inferences: *buys*(*x*, *y*) ⇒ *owns* (*x*, *y*) and *gives* (*x*, *y*, *z*) ⇒ *owns* (*y*, *z*).

**Fig. 1:**
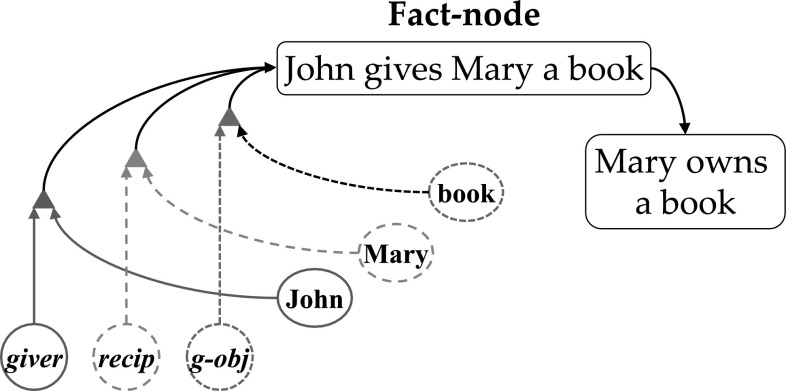
Variable binding based on synchrony of activation in Shruti (based on Shastri and Ajjanagadde, 1993, Figure 12). The nodes for arguments and thematic roles are in synchrony: *John* with *giver* (*green*, *unbroken lines*), *Mary* with *recipient* (*red*, *long-dashed lines*), and *book* with *given*-*object* (*blue*, *short-dashed lines*). Synchronous nodes activate coincidence detectors (*triangles*), which activate a proposition detector (‘fact node’) for *John gives Mary a book*. A reasoning process can then activate *Mary owns a book* (recip = recipient, g-obj = given-object). (Color figure online)

In Fig. 1 we represent a part of the connection circuit in Figure 12 of Shastri and Ajjanagadde (1993) that instantiates the inference that if *John gives Mary a book* then *Mary owns a book*. In this connection circuit there are item nodes for *John*, *Mary* and *book*, and thematic relation nodes for *giver*, *recipient* (*recip*) and *given*-*object* (*g*-*obj*). There is also a ‘fact node’ (F1 in Figure 12 of Shastri and Ajjanagadde 1993) or ‘collector node’ (Wendelken and Shastri 2004) for the particular fact or belief *John gives Mary a book*. The activation of this fact node is essential for making the inference *Mary owns a book*. The role of synchrony is to ensure the selective activation of this fact node. So, in Fig. 1 the fact node for *John gives Mary a book* is activated because the activation of *John* is in synchrony with *giver*, the activation of *Mary* is in synchrony with *recipient* and the activation of *book* is in synchrony with *given*-*object*.

Because the synchrony relations in Fig. 1 uniquely apply to the fact node *John gives Mary a book*, this fact node is activated, instead of other fact nodes, such as *Mary gives John a book*. Notice, however, that the fact node for *Mary gives John a book*, and the dedicated connection structure between this fact node and the item and thematic relation nodes, would be needed to make the inference if *Mary gives John a book* then *John owns a book*. Thus, dedicated connection structures and fact nodes as illustrated in Fig. 1 are needed for any specific inference that Shruti can make. This turns Shruti into a connectivity based binding model, in contrast to Feldman’s assumption.

Other examples of specific (high-level) nodes and dedicated connection structures for producing behavior in synchrony based binding models are the use of role-filler nodes, e.g., *Bill*-*lover*, *Mary*-*knower*, *Mary*-*beloved*, and proposition nodes, e.g., *loves(Bill, Mary)* and *knows(Mary, loves(Bill, Mary))*, in the Learning and Inference with Schemas and Analogies (LISA) model (e.g., Hummel and Holyoak 2003) and the use of role-filler nodes, e.g., *larger*-*Fido*, *smaller*-*Sara*, and proposition units, e.g., *bigger(Fido, Sara)*, in the Discovery of Relations by Analogy model (DORA) of Doumas et al. (2008).

**Fig. 2:**
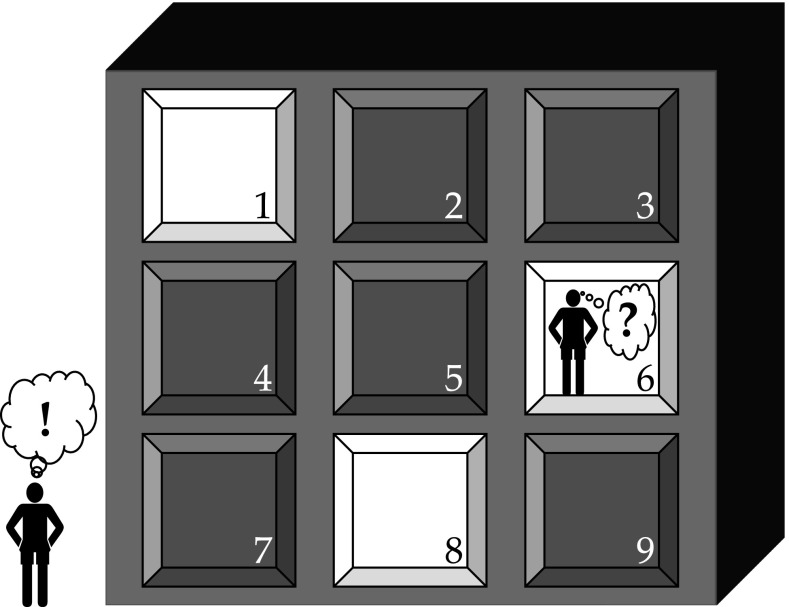
A difference in perspective, or frame of reference, can result in different information obtained about a situation. Here, an office building in which some of the rooms are lit in the evening, with two frames of reference: an outside observer in front of the building and an inside observer in one of the rooms

The use of proposition representations in the LISA model is described by Knowlton et al. (2012, p. 374): “LISA codes an analog by binding distributed representations of roles to distributed representations of their fillers (…). For each individual analog, a hierarchy of localist structure units represents objects (O), relational roles (R), individual role bindings (RB), and complete propositions (P).” Figure 2 in their paper makes this very clear also. And it illustrates that synchrony is used to effectively using these representations.

So, in each of these cases we see that synchrony models use available connection structures and representations to produce behavior. In fact, the examples given show that these synchrony models rely on very specific circuits representing very specific combinations of items, up to specific representations of entire propositions as *John gives Mary a book* in Fig. 1 or even hierarchical (nested) propositions as *Mary knows that Bill loves Mary* in LISA. The role of synchrony in these models is not to avoid these specific circuits and representations but to activate them selectively. In Fig. 1, the synchrony between the item nodes and the thematic relations nodes results in the activation of the fact node for *John gives Mary a book* instead of any other fact node (proposition representation). But this selective activation process requires the existence of these representations and the connection structures activating them in the first place.

Two remarks should be made here. Firstly, the fact that models like Shruti and LISA use proposition representations in their operations is not an issue, because these models are models of processes based on long term memory. Obviously, proposition representations can (and will) be a part of long term memory. The use of proposition representations in these models becomes an issue only when these models are seen as solutions for the problem of novel variable binding, as in Feldman’s (2013) analysis. Secondly, just like the ‘static’ (connectivity based) models, label (synchrony) based models of binding appear to rely on existing connection structures as well when they produce behavior.

Hence, the question arises of why Feldman (2013) believes that the two types of models are different in the use of existing connection structures, and why he believes that a model like Shruti could handle novel variable binding, even though it crucially depends on proposition representations. In our view, this results from a fundamental error in analyzing the role of synchrony of activation in binding models, as we will outline below.

## Synchrony and frame of reference

An essential aspect of the synchrony of events is that it is relative to the frame of reference in which the events are observed. That is, two events can be synchronous in one frame of reference but not in another (e.g., Misner et al. 1973). Thus, to assess the role of synchrony of activation in binding models we have to consider the frame of reference in which it is observed (the need to identify the frame of reference in which observations are made is one of the basic tenets of physics since Galilei).

Figure 2 illustrates an example. Assume an office building where some people are still working in the evening, which requires them to lighten their rooms. As observed by an outside observer (e.g., standing on the pavement in front of the building), this occurs in three of the rooms in the building (1, 6 and 8). This observer might come to the conclusion that the people in these rooms are working together (are ‘bound’ so to speak) because of their simultaneous presence in the evening. But what about an observer located in one of the rooms, say room 6? Would this person know that people are also working in room 1 and 8, but not in the other rooms?

The key point here is that this observer would not be able to know that in the same manner as the outside observer. The frame of reference for the latter is different from the frame of reference of an inside observer. The outside observer overlooks all rooms, so he or she can directly see which rooms are lit. The inside observer cannot directly see that. Of course, he or she could try to obtain that information, e.g., by making contact with the other rooms. But this requires a process not needed for the outside observer. This process could influence the observations made by the inside observer (e.g., because there are no connections with certain rooms, or because connections cause a delay in contact). Yet, to conclude that the people in the building are working together, we need the perspective of the inside observers. The perspective of an outside observer does not suffice to reach that conclusion.

**Fig. 3:**
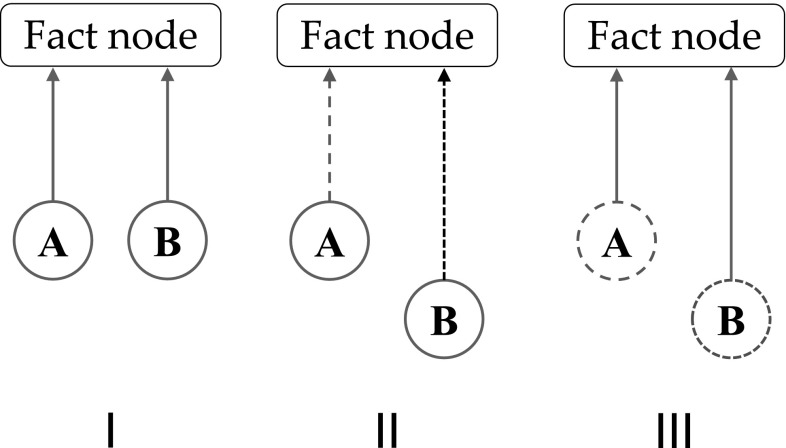
The importance of the frame of reference in analyzing synchrony of activation. In situation I, two source nodes (*A*, *B*) are in synchrony (*red*, *unbroken lines*) in an outside frame of reference. They are also in synchrony in the frame of reference of the target (Fact) node, because their activation arrives in synchrony (*red*, *unbroken lines*). In situation II, *A* and *B* are in synchrony in the outside frame of reference (*red*, *unbroken lines*), but not in the frame of reference of the Fact node, because their activation does not arrive in synchrony (*green* and *blue*, *dashed lines*). In situation III, *A* and *B* are in not synchrony in the outside frame of reference (*green* and *blue*, *dashed lines*), but they are in synchrony in the frame of reference of the Fact node, because their activation arrives in synchrony (*red*, *unbroken lines*). (Color figure online)

So, we need to identify the frame of reference when we assess the role of synchrony of activation in binding models. Feldman (2013, p. 6b) described the role of synchrony of activation in binding as follows: “When an attribute node fires in-phase with an object node, this coincidence represents a binding between them.” However, this statement is meaningless because it does not identify the frame of reference in which the synchrony of activation is observed or analyzed. The same is true of Figs. 2 and 3 presented by Feldman (2013). These figures indicate the synchrony of activation (or lack thereof) between rows or nodes referring to the variables in a binding process. These figures are also meaningless because they do not identify a frame of reference as well. When we look at Fig. 1 in this paper we can see a similar situation. We might conclude from this figure that the node for *John* is bound with the node for *giver*, because of their synchrony of activation. However, this statement is also meaningless unless we specify the frame of reference.

But perhaps a frame of reference is implicit in Feldman’s description quoted above and the Figs. 2 and 3 in Feldman (2013). Comparing them with the situation illustrated in (our) Fig. 2, it seems quite clear that Feldman’s description and figures are made in the outside frame of reference. This can also be deduced from the description given by Feldman (p 6b): “Fig. 2 below shows an example of temporal phase binding… Notice that the triangles (denoting spike trains) in row 1 remain aligned with those in row 5 and similarly for rows 3 and 6.” As the word ‘notice‘ indicates, a reader can see the phase synchrony between rows 1 and 5 and that between rows 3 and 6 in the figure referred to by Feldman. But as readers we have an outside frame of reference, which allows us to see the phase synchrony in the rows of Feldman’s Fig. 2, or the nodes in his Fig. 3, just as we can see the phase synchrony between *John* and *giver* in Fig. 1 presented here. It is important to note that a researcher measuring brain activity also obtains observations in an outside frame of reference. In that frame of reference one can indeed observe two neurons firing with the same frequency or firing in phase synchrony.

But an outside frame of reference is not the frame of reference in which the brain operates. So, by analyzing a problem in an outside frame of reference we might begin on the wrong foot. For example, we might assume that the problem has been solved when in fact it hasn’t. Dennett (1991) made a similar point when he discussed Crick and Koch’s view of the role of frequency synchronization for consciousness. In his words (Dennett 1991, p. 255):

“Here, for instance, is a hypothesis hazarded by Francis Crick and Christof Koch:

We have suggested that one of the functions of consciousness is to present the results of various underlying computations and that this involves an attentional mechanism that temporarily binds the relevant neurons together by synchronizing their spikes in 40 Hz oscillations …

So a function of consciousness is to *present the results of underlying computations*—but to whom? (…) Crick and Koch do not go on to ask themselves the Hard Question: *And then what happens*? (“And then a miracle occurs”?).

Thus when we observe phase synchrony in an outside frame of reference (e.g., by measuring brain activity in a laboratory) we have to ask ‘Then what happens?’ by analyzing how phase synchrony is used by the brain. For this, we have to analyze the problem from an inside frame of reference. This in turn raises the question of what this frame of reference, i.e., the frame of reference used by the brain, is. Feldman (2013) does not give us that information.

So, we have to guess. For example, we could analyze the binding problem depicted in Fig. 1 presented here from the perspective (frame of reference) of one of the nodes, say the node for *John*. In the outside perspective we see that it is in (phase) synchrony with the node for *giver*. Yet, the question is whether the node for *John* itself would be able to ‘know’ or ‘see’ that it is activated in synchrony with the node for *giver*. To answer this question we have to identify a mechanism that allows the node for *John* to arrive at this conclusion. However, neither Feldman (2013) nor the synchrony based binding papers we analyzed above provide such a mechanism.

Possibly the question of whether a node (neuron) would be able to ‘know’ or ‘see’ that it is activated in synchrony with another node (neuron) is the wrong question. Perhaps the solution would be that no single node (neuron) would have to observe synchrony of activation for binding to occur. Instead, only the ‘overall’ system (i.e., the brain) would have ‘know’ or ‘see’ that. But this solution in fact just rephrases the problem: how would the ‘overall’ system (brain) be able to know or see that? To answer this question we need the description of a mechanism that shows how the information (synchrony of activation in this case) can be used by the brain. Without such a mechanism we would indeed be relying on some kind of miracle to occur.

Possibly this mechanism is already available. Above we illustrated and analyzed that synchrony based binding models rely on specific connection structures to produce behavior, as illustrated in Fig. 1. These connection structures would provide an inside frame of reference as used by the brain, and thus an inside frame of reference in which we could (and have to) analyze the role of synchrony of activation in variable binding.

As illustrated in Fig. 1, in Shruti the fact node for *John gives Mary a book* is activated when *John* is in synchrony with *giver*, *Mary* is in synchrony with *recipient* and *book* is in synchrony with *given*-*object*. So, in Shruti the inside frames of reference for observing synchrony of activation are given by the fact nodes of the model (or by the coincidence detectors that activate the fact nodes, as in Fig. 1). This, of course, is in agreement with the roles these fact nodes play in producing behavior. It is also in agreement with the role that synchrony of activation plays in the model: the selective activation of fact nodes. A similar observation can be made for the other synchrony based binding models we analyzed above.

An inside frame of reference as given by the fact nodes in Shruti allows us to analyze the role of synchrony of activation in binding models, and compare inside and outside frames of reference in this respect. Figure 3 illustrates the binding of two source nodes, A and B, in three different situations. In situation I, A and B are in (phase) synchrony in the outside frame of reference. They are also in synchrony in the frame of reference of the fact (or collector) node to which A and B are connected, because the connection paths between the source nodes and the fact node are of equal length. So the activation from A and B reaches the fact node at the same time (assuming equal conductivity along both paths). In situation II, A and B are again in synchrony in the outside frame of reference, but not in the inside frame of reference, because the paths between the source nodes and the fact node are of different length. This prevents the activation of the fact node. In situation III, A and B are not in synchrony in the outside frame of reference, but they are in synchrony in the inside frame of reference, because the difference in path length between the source nodes and the fact node ‘compensates’ the asynchrony between A and B (as seen in the outside frame of reference). Because the system in Fig. 3 operates in the inside frame of reference, it detects binding by synchrony in situations I and III, but not in situation II. In the outside frame of reference, however, A and B are in synchrony in situations I and II but not in III.

Figure 3 underlines the difference between an outside and inside frame of reference as illustrated in Fig. 2. Figure 3 (again) illustrates that synchrony based binding models need specific connection structures and representations to achieve binding in the inside frame of reference. Furthermore, Fig. 3 also shows another demand that synchrony based binding models have to fulfill. Not only do they need specific connection structures and representations to achieve binding (and produce behavior on the basis of binding), but these connection structures have to be of a restrictive kind. They have to ensure that the synchrony of activation of the (source) nodes is maintained at the fact or collector nodes that detect the synchrony between the sources nodes, as with *John*, *giver* and *John gives Mary a book* in Fig. 1. Because we are dealing with the need for arbitrary variable bindings these requirements have to be fulfilled for all possible combinations of source nodes and fact nodes.

So, both ‘static’ (connectivity based) models and ‘label’ (synchrony) based models of binding appear to rely on existing connection structures when they produce behavior. This would also be true when these models could handle novel variable binding. In this sense, both types of models are connectivity based.

This raises the question of how novel variable binding, or novel combinations of constituents, could be represented and processed in existing connection structures. The specific binding mechanism involved, e.g., dynamic interaction or synchrony of activation, could vary. But any given mechanism cannot depend on the existence of (entire) proposition representations in dealing with novel variable binding.

## Small-world connection structure

Connectivity based models can be models of variable binding when their connection structure resembles a small-world network. A small-world network is a connection structure that combines dense local connectivity with sparse long-range connectivity in such a way that the average path length between any two nodes in the network is low (Watts and Strogatz 1998). As an example, consider the collection of airfields visited by airlines. Any two airfields in this set are connected by a flight. Not because there is a direct flight between any two fields, which would indeed be very ineffective and inflexible, but because the set resembles a small-world network. From any local field you can go to, say, a national hub and from there to a continental hub. This combines dense local connectivity with sparse (long-range) connectivity. As a result, you can travel from any field to any other with just a few transfers (which gives low average path length).

There is strong evidence that the connectivity structures of brains, including the human brain, resemble a small-world network structure (see Shanahan 2010, for a review and discussion). In this way (temporal) connection paths can be created that provide the basis for variable binding. Here, we do have to take into account the specific nature of the process at hand. That is, variable binding in human cognition is productive and flexible, but it does satisfy certain boundary conditions.

As an example, consider the phonological structure of words. At birth, babies are ‘universal speech perceivers’ in the sense that they are sensitive to the phonetic contrasts in all natural languages (e.g., Doupe and Kuhl 1999). But at the end of the first year, they have become sensitized to their own specific natural language. At that time they recognize language specific phonetic combinations but fail to distinguish between contrasts in other languages. Thus, it would seem that the development of speech and language recognition results in a ‘neural commitment’ (Kuhl 2004), in which the brain has developed neural circuits that can recognize *and combine* phonetic units of the familiar language but fail to do so for unfamiliar languages.

This ‘neural commitment’ of the brain that develops through learning and growth forms a boundary condition for variable binding. In the case of phonology, natural language users would be able to perceive and produce new word forms in their own language (e.g., ‘Jabberwocky’), but not in unfamiliar languages. In terms of connectivity based models of variable binding, this difference results from the fact that natural language speakers have developed the neural circuits and connection structures that allow them to recognize and combine the phonetic units of their natural language. So, a model of variable binding would have to account for that. But that model would not have to account for universal phonetic variable binding, based on the phonetic units of all languages. This difference puts a limitation of the flexibility of variable binding and gives a boundary condition for binding models.

With this caveat in mind we can look at a few examples of connectivity based binding. The first example is episode binding. We have the ability to bind arbitrary items (e.g., persons, objects, events) as belonging to an episode. This is clearly a very important cognitive ability, which allows us to deal with complex and changing environments. An arbitrary episode in our environment could consist of a new collection of familiar items, never encountered before in that specific combination. But with episode binding we can reproduce which items co-occurred in an episode.

The hippocampus and the surrounding medial temporal cortex play an important role in this ability. Norman and O’Reilly (2003) proposed and simulated a model that accounts for a range of behavioral observations related to episode binding. The model is based on the anatomical structure of the hippocampus and its connections with the neocortex. These connections resemble a small-world network structure. The hippocampus connects to the medial temporal cortex (the entorhinal cortex). In turn, the medial temporal cortex is connected to a broad range of cortical regions in the frontal, temporal and parietal lobes (e.g., Squire and Wixted 2011).

The connection structure between the hippocampus and the neocortex forms a loop. Connections enter the hippocampus from the entorhinal cortex. They connect to the dentate gyrus and CA3 of the hippocampus. From there, connections pass on to CA1 of the hippocampus and back to the entorhinal cortex, and then to the neocortex. The connections between the entorhinal cortex and hippocampus and within the hippocampus exhibit long-term potentiation (LTP), by which these connections are rapidly modified (strengthened). For example, early LTP results from a single train of action potentials and last for 1–3 h. Repeated activation results in late LTP that can last for at least a day (e.g., Kandel 2001).

**Fig. 4:**
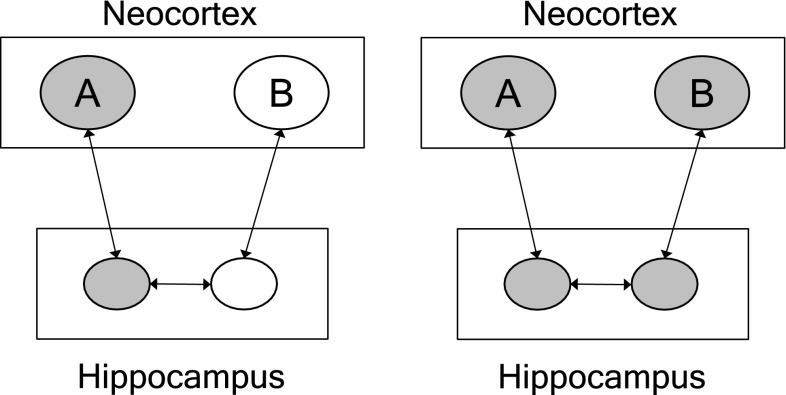
Network structure in episode binding. Two arbitrary items (object, events) *A* and *B*, represented in the neocortex, are bound in an episode via connections and rapid long-term potentiation in the hippocampus and surrounding medial temporal lobe (after Norman and O’Reilly 2003). Ovals represent neurons (or populations of neurons). Gray ovals are active. When *A* is activated in the neocortex, it will activate *B* through the (temporal) connection structure between them in the hippocampus (and medial temporal lobe)

The combination of (early) LTP and the connection structure resembling a small-world network provide the basis for arbitrary episode binding. Figure 4 schematically illustrates the binding of two arbitrary items (e.g., persons, objects, events) A and B that co-occur in an episode. The neurons representing (processing) the items somewhere in the neocortex are connected to the hippocampus (via the medial temporal cortex). These connections are sparse in the sense that two different items activate substantially different sets of neurons in the hippocampus, which results in pattern separation (Norman and O’Reilly 2003). LTP ensures a (temporal) strengthening of these connections so that both items are temporarily stored in memory. However, connections within the hippocampus (CA3) are also strongly recurrent. With LTP this results in the (temporal) interconnection between the representations of A and B in the hippocampus. As a result, A and B are bound in an episode.

The effect of episode binding can be seen in the pattern completion capability of the hippocampus. Due to the recurrent connections, the hippocampus can restore an entire pattern when only a part of it is initially activated (Norman and O’Reilly 2003). In the left panel of Fig. 4, for example, only item A is activated in the neocortex. It will activate the set of neurons in the hippocampus to which it is (temporally) connected. In turn, these neurons activate the other set of neurons belonging to the episode, due to the recurrent connectivity (and early LTP) in the hippocampus. This, in turn, activates the item B in the neocortex, as illustrated in the right panel of Fig. 4.

In this way, the brain can bind arbitrary items in an episode. There is substantial empirical evidence related to this model of binding, as partly outlined above. Another example of evidence are the ‘concept’ cells found in the medial temporal lobe (e.g., Quian Quiroga 2012). These cells were found in the medial temporal lobe (including the hippocampus) of epilepsy patients, using intracranial measurements. A remarkable feature of these cells is their highly selective and multimodal invariant response profile. This indicates that these cells receive activation from different regions in the cortex, involved in different forms of information processing (e.g., recognizing pictures vs. recognizing names), in line with a small-world network structure. The concept cells are ideally suited for rapid episodic memorization and association (Quian Quiroga 2012), as illustrated with cells in a patient that responded selectively to the experimenters (persons) involved in the experiments. Clearly, it would be of importance for an observer (a patient in this case) to rapidly ‘bind’ important individuals to an episode (e.g., an experiment) the observer is involved in. In other words, concept cells also seem to rapidly register (bind) important aspects of a here and now situation.

Recently, Wang et al. (2014) tested how the hippocampus could “serve as a ‘hub’ to support binding of information from distinct processing modules into associative memories” (Wang et al. 2014, p. 1054). Firstly, they determined a specific connection path between the hippocampus and a part of the cortex (the lateral parietal cortex), using resting state fMRI. Then, they enhanced the connectivity in this path with repetitive transcranial magnetic stimulation. After this, resting state fMRI indeed showed an increased connectivity between the hippocampus and the selected cortical area. Behavioral experiments showed that the enhanced connectivity resulted in an improved memorization of arbitrary and novel pairs of items, consisting of faces and written words.

The results of the Wang et al. (2014) experiment show that an existing connectivity structure can be used to associate (bind) novel combinations of items. This ability can even be manipulated by enhancing the connection structure. The effects of this memorization lasted up to 24 h, which indicates that they are based on forms of synaptic enhancement.

## Synchrony models with small-world connection structure

Small world connection structures could be combined with synchrony of activation. For example, Li and Li (2011) investigated how synchrony of activation could enhance input segmentation in a small world network structure.

Baars et al. (2013) discussed the role of synchrony of activation and resonance in forming functional hubs in the Global Workspace model of consciousness. Their model includes a small world network structure consisting of the hippocampus and surrounding areas, as illustrated in Fig. 4. Other structures of this kind in their model are the cortical thalamic pathways. Baars et al. (2013, p. 20) describe the connection structure in this pathways and the role of synchrony (oscillations) as follows: “Cortico-thalamic pathways run in all canonical directions and follow small-world organization, so that each array is efficiently linked to many others. The entire system acts as an oscillatory medium, with markedly different global regimes in conscious and unconscious states.”

In these examples it is clear that the underlying (small network) connection structure is an existing structure. So, again, as in the examples of binding models discussed above, the role of synchrony in these models is not to avoid specific connection structures but to use them effectively. A similar observation can be made of synfire chains. They also depend on existing network structures, so they do not violate the assumption that connection structures are needed for binding. But as Abeles (2009) has pointed out they could also achieve forms of combinatorial binding.

Although we are not aware of models of novel variable binding along the lines as presented in this section, it is clear that such models would have to account for novel variable binding using an existing connection structure, contrary to Feldman’s assumption, and they have to do so without relying on entire proposition representations.

## Binding by process

Another option for using small world connection structures in novel variable binding is a form of binding we refer to as ‘binding by process’. To illustrate what this means, consider again the set of airfields visited by airlines. Any two airfields in this set can be bound. Not by attaching a common label to them (which then has to be observed, say from space). Instead, any two airfields are bound when a person flies from one to the other, using the flexible network structure in the set. The binding of items A and B in Fig. 4 is of the same nature. They are not bound by some common label. Instead, they are bound because activation can flow from one to the other. This is possible because a (temporal) connection path is formed in the flexible connection structure to which A and B belong.

Examples of binding by process are the model of episode binding illustrated in Fig. 4, binding of perception and action (Zylberberg et al. 2010), binding in visual working memory (Swan and Wyble 2014) and binding in processing relational knowledge (Pinkas et al. 2012).

Binding by process is also the basis of our model of variable binding (van der Velde and de Kamps 2006a, b).

We can only briefly outline this model here. In (van der Velde and de Kamps 2006a, b, 2010) we showed in detail how neural mechanisms can integrate arbitrary words in the lexicon in arbitrary sentence structures, including novel sentences.

**Fig. 5:**
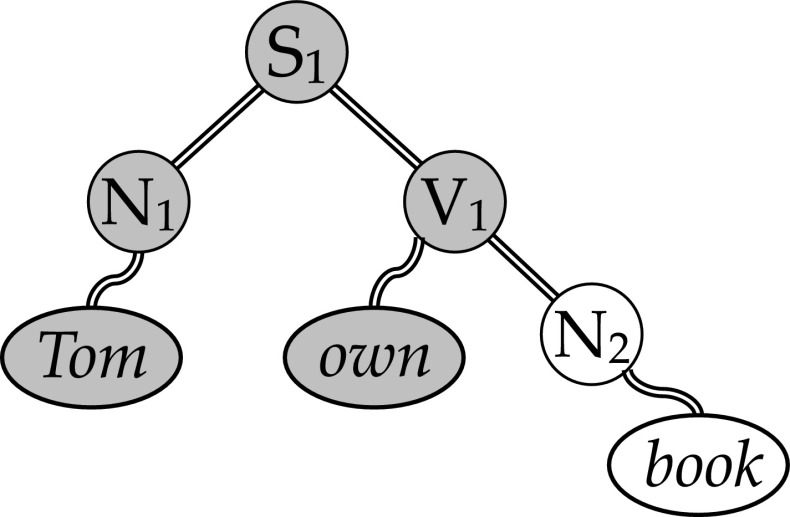
Binding of the words *Tom*, *own* and *book* in the neural blackboard representation of the sentence *Tom owns (a) book* (after van der Velde and de Kamps 2006a, b). The *ovals* represent neural word representations, the *circles* represent ‘syntax’ populations in the neural blackboard. S_1_ is a ‘sentence’ population, N_1_ and N_2_ are ‘noun’ populations, V_1_ is a ‘verb’ population. The *gray ovals* and *circles* are activated by the question “What does Tom own?”

The model is a connectivity based binding model in which neural word representations can be temporarily bound in a sentence context, as illustrated with the binding of the neural word representations of *Tom*, *own* and *book* in *Tom owns* (*a*) *book* in Fig. 5. To achieve this in a flexible manner, a small-world like connection structure is needed. We referred to this structure as a ‘neural blackboard’. Furthermore, the binding has to be relational. In Fig. 4, the binding between A and B is in the form of an association, but that is not sufficient for binding words in a sentence.

To achieve relational binding, the connection structure in the neural blackboard consists of ‘conditional connections’, illustrated with the double-line connections in Fig. 5. These connections consists of gating circuits that need to be ‘opened’ or ‘activated’ to allow activation to flow. In the model, conditional connections are activated when certain conditions are met. For example, by the (temporal) activation of a neural population that operates as a working memory for a particular binding (e.g., binding the word representation of *Tom* to a neural population *N*_*1*_ in Fig. 5, for details see van der Velde and de Kamps 2006a, b).

The small-world like connection structure illustrated in Fig. 5 allows all familiar words to be bound in arbitrary (but regular) sentence structures. This can occur because all nouns for example are connected to a limited set of N_i_ populations (referred to as ‘noun assemblies’). When the conditional connection between a noun and a particular N_i_ assembly is activated, they are temporarily bound. In a similar way, verbs can be bound to verb assemblies (V_i_). In turn, the specific N_i_, and V_i_ assemblies can be bound in a specific sentence structure (which can include other word types as well). Because binding is temporal (determined by the ongoing or ‘delay’ activation in the neural population that controls the specific conditional connection), sentence structures will decay over time in the neural blackboard, allowing the connection structures in the blackboard to be used for other sentences.

Binding by process is illustrated in Fig. 5. The gray structure illustrates the sentence part in the blackboard that is activated by the question “What does Tom own?”. The answer to this question can be found by activating the conditional connection between V_1_ and N_2_. This connection can be activated because the question asks for the theme (object) of the verb in the sentence, and V_1_ and N_2_ are connected as verb and theme. Thus, this connection is activated by a control signal that activates the verb-theme relations in the blackboard (for a simulation of this process, see van der Velde and de Kamps 2006a, b). This will activate N_2_ and with it *book*.

Above we argued that all binding models must be connectivity based models, because a connection structure is needed to produce behavior, that is, interconnect sensory and motor activation. The binding by process illustrated in Fig. 5 (and Fig. 4) is of this kind. In both cases the binding is achieved whenever a behavior (output) needs to be produced that depends on the binding relation. In Fig. 5 this consists of answering the question “What does Tom own?”. In Fig. 4 this consists of answering a query like “What item co-occurred in the episode to which A belongs?”. In other words, the binding by process in connectivity based models is a direct consequence of the need for connection structures to produce behavior.

## Novel variable binding in existing connection structures

Feldman (2013) argued that connectivity based binding models cannot handle novel variable binding. The model illustrated in Fig. 5 and described in detail in (van der Velde and de Kamps 2006a, b, 2010) can combine any word in the lexicon in any arbitrary sentence context. That is, it can handle the ‘Miller set’ (see section “Variable binding in novel combinations”). Because of the magnitude of this set, most of these sentences are novel. Moreover, the architecture illustrated in Fig. 5 does not rely on representations of entire propositions. So, novel variable binding is possible with an existing (small world like) connection structure.

However, Feldman used the ‘Sonnet’ sentence as an example of novel variable binding (see above), in which he introduced a new word as well (i.e., the name ‘Sonnet’). So, we can also bind a new word or name, such as *Sonnet*, in sentences or inferences like if *John gives Sonnet a book* then *Sonnet owns a book*. These sentences are not in the Miller set because that set is based on an existing lexicon. Instead, they belong to the ‘extended Miller set’ (see above).

It is quite remarkable to see that Feldman proceeds, after introducing the issue of novel variable binding with the ‘Sonnet’ sentence, with discussing and advocating binding by synchrony, in particular Shruti (Shastri and Ajjanagadde 1993). In particular, he does not raise the question as to whether these models (e.g., Shruti) could handle new words in sentences or inferences like if *John gives Sonnet a book* then *Sonnet owns a book*. Apparently he assumes they can. Perhaps because a new (but apparently available) node representing *Sonnet* could be in synchrony with the nodes for *recipient* or *owner*, which would assure their binding. But, as we discussed above, this is binding in an outside frame of reference, which is not the correct frame of reference for analyzing neural binding problems.

In Fig. 1 we illustrated that in Shruti an inside frame of reference for binding by synchrony is given by the fact (or collector) nodes that are needed to produce behavior (make the inference). So, for the inference that if *John gives Sonnet a book* then *Sonnet owns a book*, we need a fact node for *John gives Sonnet a book*. And we need the dedicated connection structure that produces the activation of this node based on the synchrony relations *John*-*giver*, *Sonnet*-*recipient* and *book*-*given*-*object*. In fact, we need fact nodes and dedicated connection structures for all potential relations in which *Sonnet* could appear. Shruti does this for inferences in long term memory. But if *Sonnet* is a new node, where do all of these fact nodes and connection structures come from?

The issue of binding new items is of course also important for binding models. We briefly dealt with this issue in our target paper (van der Velde and de Kamps 2006a, p 61a) and more extensively in the reply paper (section R4 and Figure R1 in van der Velde and de Kamps 2006b). The solution is based on the observation that language has (at least) a two tier structure (Jackendoff 2002). That is, words can be bound in an unlimited number of sentence structures (giving the Miller set). But words themselves are also compositional (phonological) structures, which arise by binding phonemes and morphemes in line with the phonological regularities underlying a given language. So, the reason that we have a potentially unlimited number of words (items) that could be bound to a variable in a sentence structure (giving the extended Miller set) results from the fact that words themselves are compositional structures. But the set of phonemes and morphemes we use to make words is limited in a given language.

Above we discussed the observation that babies are born as universal language perceivers, but that in the course of a year they become specialized for their own natural language. This specialization results from a ‘neural commitment’ (Kuhl 2004) that arises during development. We would argue that this neural commitment results in a small-world like connection structure that allows the formation of compositional phonological word-forms to be made and perceived. We referred to this small-world like connection structure as a neural blackboard for phonological structure (van der Velde and de Kamps 2006b).

**Fig. 6:**
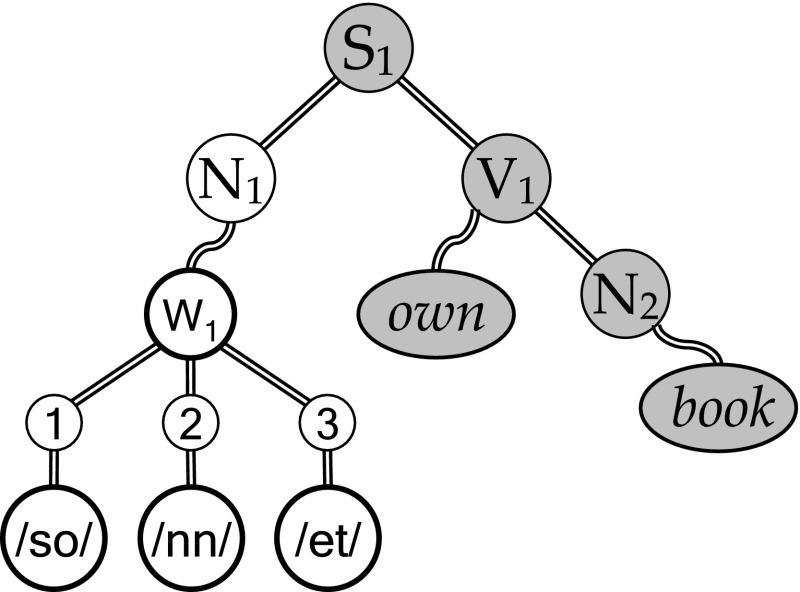
The neural sentence representation of *Sonnet owns book*, by combining a phonological neural blackboard with the neural (sentence) blackboard in Fig. 5. The phonological neural blackboard binds the familiar phonemes/morphemes /so/, /nn/ and /et/ to a ‘word assembly’ W_1_, which binds to the sentence structure *Sonnet owns book* (after van der Velde and de Kamps 2006b). The *gray ovals* and *circles* are activated by the question “Who owns the book?”

Figure 6 illustrates the interaction between the blackboard for phonological structure and the blackboard for sentence structure in establishing the binding relations in the sentence *Sonnet owns a book*. Assuming that /*so*/, /*nn*/ and /*et*/ are the phonemes/morphemes of the word *Sonnet*, they bind to a ‘word assembly’ in the phonological blackboard. In turn, this word assembly can bind to sentence structures. This binding will be regulated by the perception that *Sonnet* is a noun (van der Velde and de Kamps 2010). In a similar way as illustrated in Fig. 5, the combined blackboard will answer a question like “Who owns the book?” by activating /*so*/, /*nn*/ and /*et*/ in the phonological blackboard (based on the initial activation of the sentence part *own book*, activated by the question).

It is important to understand that the boundary conditions on binding come into play here. We have the ability to make and recognize arbitrary phonological structures in our own language, including new words or nonsense (pseudo)words like ‘Jabberwocky’. We can do this because the set of phonemes and morphemes in a language is limited. Hence, during growth and learning we can develop a small-world like connection structure that allows arbitrary phonological combinations to be made in our language. But we do not have that ability for unfamiliar languages, because we have not developed small-world like connection structures for their phonological structures. In the same way, we can form and make arbitrary sentence structures, but only in the languages we are familiar with. To do so in unfamiliar languages would require an extensive process of learning, needed to develop the small-world like connection structures for these languages.

There is clearly less experimental evidence for small-world like connection structures for language compared to episode binding, partly due to the complexities involved in studying the human brain. However, in a neuroimaging study Pallier et al. (2011) observed a difference between two sets of brain areas, using a contrast between sentences with words and sentences with (meaningless) pseudowords. One set of brain areas seemed to be involved in processing and representing the (lexical-semantic) content of the sentences. The other set was activated by the abstract (syntactical) structure of sentences, irrespective of their content. This distinction is in line with the distinction between neural word representations and a neural ‘syntax’ blackboard as illustrated in Figs. 5 and 6.

## Conclusions

We compared two types of models used for variable binding, one based on binding by synchrony and the other based on binding by available connection structures. In his review of the neural binding problem(s), Feldman (2013) argued that models based on available connection structures (or ‘static’ models as he called them) cannot account for novel variable binding but synchrony based models can.

We showed that Feldman (2013) used an outside frame of reference in his analysis of binding by synchrony, instead of an inside frame of reference in which the (variable) binding problem should be analyzed. If the latter frame of reference is taken into account, binding by synchrony also relies on existing connection structures. This is not a coincidence. Connectivity based models are the only viable candidates as models of binding because connection structures are needed to produce behavior, including behavior based on novel variable binding.

The synchrony based models analyzed by Feldman (2013) do indeed use specific representations and connection structures because they (to their credit) produce behavior. The role of synchrony in these models is not to avoid these representations and connection structures, but to use them effectively. Furthermore, the representations and connection structures used in synchrony models analyzed by Feldman (2013) are very specific, up to entire propositions and nested propositions. This is not an issue because these models are models of processes based on long term memory. But for novel variable binding, aimed for by Feldman (2013), representations of entire propositions and nested propositions will not be available.

Novel variable binding can be achieved on the basis of existing connection structures when these structures resemble that of a small-world network. Such connection structures provide the flexibility needed for variable binding, even for the binding of novel items (e.g., words, events) in novel structures (e.g., sentences, episodes). One way to achieve this is by creating a temporal connection structure in line with the cognitive structure (e.g., proposition) at hand. This temporal structure can be used to produce a flow of activation in line with the represented binding relations. This binding by process is in line with the need for connection structures to produce behavior. In also underlines the very dynamical way in which variable binding is achieved in small-world like connectivity based models.

But we have to take the boundary conditions on these forms of binding into account. The variable binding will succeed only for compositional structures for which the basis (recognizing items and the ability to form combinations) has been learned. An example is the ability to recognize and make arbitrary phonological word-forms in a familiar language, which begins to develop in the first year of life.
